# The New Economic Era Analysis of the Structure System of Chinese Household Consumption Expenditure Based on the ELES Model

**DOI:** 10.1155/2022/3278194

**Published:** 2022-08-09

**Authors:** Chaozhi Fan, Siong Hook Law, Saifuzzaman Ibrahim, N. A. M. Naseem

**Affiliations:** School of Business and Economics, Universiti Putra Malaysia, Serdang, Selangor 43440, Malaysia

## Abstract

In recent years, the new economy has entered a phase of rapid development and upgrading China's service consumption is driving the continuous optimization of the population's consumption structure. To realize the rationalization of the Chinese household consumption structure, the ELES model is used to analyze the structure system of Chinese household consumption expenditure. This article constructs the ELES model, divides the types of Chinese household consumption expenditure structure systems, establishes consumption expenditure function, analyzes the influencing factors of the consumption expenditure structure system, and obtains the analysis results from static and dynamic aspects. Based on the statistics of Chinese household consumption expenditure data in recent years, this article obtains the analysis results of the consumption expenditure structure system: the basic consumption demand and marginal consumption tendency of food are in the first place, and the consumption expenditure structure system has gradually changed into the development-type and enjoyment-type consumption mode. Through increasing the income of rural residents, guiding reasonable consumption concept, optimizing consumption environment, and so on, we can promote the proposal and implementation of the optimization of China's household consumption expenditure structure system to improve the rationalization of China's household consumption structure system.

## 1. Introduction

Consumption is not only the end of social reproduction, but also the starting point of its logic. It is also one of the three main driving forces of economic growth and plays an important role in economic growth. Consumption structure is one of the key research contents in the field of current economics. It can not only reflect the living standard and quality of residents, but also reflect the development of economy. It is a very important index in economic research. The consumption structure is affected by many comprehensive factors, which in turn affect the development of these factors [1]. Through the study of consumption structure, we can deeply understand the specific content of residents' consumption, find the characteristics of residents' consumption, and play an important role in expanding domestic demand. To study the characteristics and types of Chinese family consumption support structure is helpful to recognize the situation of China's economic development, reasonably guide the effective allocation of resources and the path of economic growth, and optimize the path of economic development.

At this stage, most foreign scholars are more committed to the construction and application of demand function model in the study of residents' consumption structure, which brings a lot of innovation in research methods. At the same time, the entry point of foreign scholars' research on the consumption structure is novel and diverse. The research results of foreign scholars on consumption function and consumption system model have laid a solid theoretical and model foundation for the research on China's household consumption expenditure structure. However, most of the domestic scholars introduce new variables to innovate based on the existing models to inject new vitality into the research of consumption structure. Based on the research results at home and abroad, we can find that the AIDS model and Panel Data are mostly used in the research of the consumption structure, while VAR model, factor analysis, cluster analysis, and grey correlation analysis are mostly used in the research of influencing factors of the consumption structure. However, these methods can only rank the influencing factors of the consumption structure, so the analysis results of the consumption expenditure system are one-sided, so the ELES model is introduced.

The ELES model is an extended linear expenditure system model, which assumes that people's demand for various goods in a certain period depends on people's income and the price of various goods. Moreover, people's demand for all kinds of goods is divided into two parts—basic demand and demand beyond basic demand, and they think that basic demand has nothing to do with income level. After the basic demand is met, residents arrange various nonbasic consumption expenditures according to certain marginal consumption tendency [2]. In the process of analyzing the Chinese household consumption expenditure structure system, the application of the ELES model is expected to improve the rationalization of the consumption expenditure structure.

## 2. Building ELES Model

According to the statistical data of the National Bureau of Statistics on the per capita annual income and expenditure of urban households, the ELES model takes the per capita disposable income of residents at time *t* as the education consumption expenditure and then adds the expenditure of cultural and entertainment supplies to other expenditures, to properly process the statistical data of the National Bureau of Statistics [3]. The basic expression of the extended linear expenditure system of China's household consumption expenditure is as follows:

(1) $$\begin{array}{c} V_{it}=p_{it}q_{it}+b_{it}\left(X_{t}-V_{0t}\right), \end{array}$$

where *t* means time, Q_*it*_ is the demand of class *i* consumption items in year *t*, *V*_*it*_ is the consumption expenditure of category *i* consumption item in year *t*, and the consumption expenditure items represented by category *i* in *V*_*it*_ have been given above; *p*_*it*_ is the price of category *i* consumption item in year *t*, q_*it*_ is the basic demand of type *i* consumption item in year *t*; b_*it*_ is the marginal propensity to consume; and *V*_0*t*_ is the total basic demand expenditure in year *t*, and its expression is as follows:

(2) $$\begin{array}{c} V_{0t}=\sum_{t}p_{it}q_{it}. \end{array}$$

In addition, *X*_*t*_ is the per capita disposable income of residents in year *t*. That is to say, the consumption expenditure of a certain kind of commodity in year *t* is equal to the sum of the basic consumption expenditure of this kind of commodity and the marginal consumption expenditure of this kind of commodity in year *t* [4]. Based on the basic model, the residual term is added to get the econometric model:

(3) $$\begin{array}{c} V_{it}=p_{it}q_{it}+b_{it}\left(X_{t}-V_{0t}\right)+\mu_{i}. \end{array}$$

After deformation and finishing the above formula, the results are as follows:

(4) $$\begin{array}{c} V_{it}=a_{it}+b_{it}X_{it}. \end{array}$$

The expression of parameter *a*_*it*_ is as follows:

(5) $$\begin{array}{c} a_{it}=p_{it}q_{it}-b_{it}V_{ot}. \end{array}$$

Then, the total basic expenditure and various basic expenditures in the *t* − *th* year are calculated as follows:

(6) $$\begin{array}{c} \left\{\begin{array}{c} V_{ot}=\sum_{i}\frac{a_{it}}{1-\sum_{i}b_{it}}\\ p_{it}q_{it}=a_{it}+b_{it}V_{ot} \end{array}\right). \end{array}$$

Therefore, we can conclude that the cross-price elasticity of Chinese household consumption expenditure is as follows:

(7) $$\begin{array}{c} \varepsilon_{ijt}=\frac{\partial Q_{it}}{\partial p_{jt}}\cdot \frac{p_{jt}}{Q_{it}}=-\frac{b_{it}p_{jt}q_{jt}}{V_{it}}. \end{array}$$

The self-price elasticity of Chinese household consumption expenditure demand is

(8) $$\begin{array}{c} \varepsilon_{iit}=\frac{\partial Q_{it}}{\partial p_{it}}\cdot \frac{p_{it}}{Q_{it}}=-\frac{b_{it}\left(X_{t}+p_{it}q_{it}-V_{ot}\right)}{V_{it}}. \end{array}$$

In the same way, we can also get the basic demand expenditure of *i* commodity.

## 3. Analysis on the Structure System of Chinese Household Consumption Expenditure

### 3.1. Classification of Consumption Structure Types

The consumption demand of Chinese families is constantly showing the characteristics of diversification, the scope of consumption expenditure is gradually expanding, new consumption hot spots are constantly emerging, and the consumption structure of residents is also showing the characteristics of multilevel. According to the different levels of meeting consumption demand, consumption structure can be divided into survival data, development data, and enjoyment data [5]. Survival materials are the materials that meet people's most basic needs, that is, the necessary food, clothing, and housing for rest to meet people's basic needs of eating, wearing, and living. They are the materials that are necessary to maintain the simple reproduction of labor force and the physical and mental strength of workers. Development materials are the materials that meet the needs of human physical and intellectual development. To make people's physical and intellectual development in an all-round way, residents need to have the necessary living materials for education, science and technology, culture, health care, and social interaction. Means of enjoyment are the material and spiritual means of satisfying people's needs for enjoyment. They include high-end consumer goods and materials necessary for people's spiritual life, such as entertainment and tourism [6]. Survival data are the most basic consumption data, and its elasticity is the smallest; development data and enjoyment data are higher level consumption data. In the Statistical Yearbook published by the National Bureau of Statistics, consumption expenditure is divided into eight categories according to the specific forms of consumption, including food, clothing, housing, family equipment, and transportation. From the perspective of the development level of consumption structure, the consumption structure can be divided into four types, as shown in Table 1.

### 3.2. Establishing Chinese Family Consumption Function

The consumption function is mainly used to measure the relationship between consumption and income. Assuming that consumption is mainly determined by permanent income, permanent income is the expected long-term income. *Y*_*p*_ is the permanent income. The expression is as follows:

(9) $$\begin{array}{c} Y_{p}=\theta Y+\left(1-\theta \right)Y_{-1}, \end{array}$$

where *Y* is the current income, *Y*_−1_ represents past revenue, *θ* is a constant, and the parameter value range is [0, 1]. Then, the form of consumption function is as follows:

(10) $$\begin{array}{c} C=kY_{p}. \end{array}$$

The persistent income hypothesis suggests that rational consumers make consumption decisions to maximize their effects, not based on temporary income in the present period. Instead, consumption decisions are based on the level of income that can be maintained long-term, that is, the level of lasting income. The life cycle hypothesis relates consumption to lifetime income and property. It assumes that consumers are rational and able to use their income sensibly and consume. The only goal of consumer behavior is to maximize utility. The life cycle and persistent income hypotheses are highly similar, emphasizing that consumption is mainly determined by future income [7].

### 3.3. The Main Influencing Factors of Consumption Structure

There are many factors affecting the consumption structure of urban residents, which can be divided into macro- and microfactors, such as industrial structure, consumer prices, and family population; they can also be divided into economic and noneconomic factors, such as income, industrial structure, and social and cultural, scientific, and technological progress [8]. These factors do not exist alone but restrict and influence each other and work together on the consumption structure. Among them, the income of residents is the most important and direct economic factor. Because income constitutes the economic basis of residents' consumption demand, the amount of income directly affects the level of residents' consumption expenditure and the proportion of all kinds of goods or services consumption, namely, consumption structure [9]. Industrial structure and consumption structure promote each other. The industrial structure determines the product structure and then the consumer goods structure, which is further reflected in the consumption structure of residents. With the increase in income, people will put forward higher consumption demand. The industrial structure is constantly adjusted and optimized to meet the gradually upgraded consumption demand through the change of product structure, thus forming a virtuous cycle. In addition, consumption environment refers to the external and objective factors that consumers face in the process of survival and development and have a certain impact on consumers, including natural environment and social environment [10]. The natural environment includes sunlight, water, air, soil, and other natural conditions for human survival; the social environment includes infrastructure, consumption and circulation market, institutional environment, and other factors.

### 3.4. Analysis on the Structure System of Chinese Household Consumption Expenditure

Under the ELES model, through the analysis of the influencing factors and components of China's household consumption expenditure structure system, the final analysis results of China's household consumption expenditure structure system are obtained from the static and dynamic aspects, respectively, and the basic demand and marginal consumption tendency of China's household consumption expenditure structure system are shown in the results.

## 4. An Empirical Analysis of the Structure of Household Consumption Expenditure in China

### 4.1. Basic Needs Analysis

According to the definition of the consumption structure in the Statistical Yearbook, consumption expenditure is divided into food consumption, clothing consumption, residential consumption, household equipment and services consumption, medical and health care consumption, transportation and communication consumption, education, culture and entertainment services consumption, and other goods and services consumption, a total of eight consumption expenditures [11]. This classification method has been adopted since China Statistical Yearbook, and the specific data of consumption structure starting from 2015 are sorted out, as shown in Table 2.

**Figure 1:**
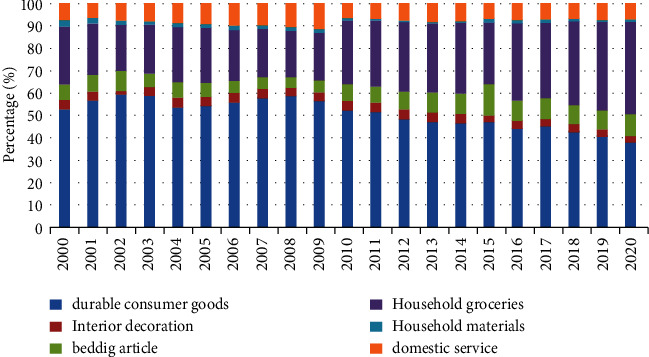
Internal structure of consumption expenditure on daily necessities and services.

The internal structure of household goods and services consumption expenditure is shown in Figure 1.

On this basis, the analysis results of the Chinese household consumption expenditure structure system are obtained from static and dynamic aspects.

### 4.2. Results of Structural Static Analysis

#### 4.2.1. Consumption Level

**Figure 2:**
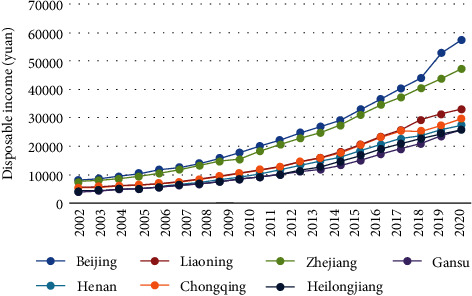
Trend of per capita disposable income in different regions.

Consumption level refers to the scale and level of life consumption and services used by individual consumers or the whole society in a certain period. The level of consumption is determined by social development and people's living standards [12]. According to the consumption situation of multiple regions, the trend of per capita disposable income of consumption level is obtained, as shown in Figure 2.

It can be seen from Figure 2 that great changes have taken place in China's household consumption level in the past two decades. The per capita disposable income has increased from 4091 yuan in 2001 to 25763 yuan in 2020, an increase of about 6.3 times. In the past two decades, the growth rate of per capita disposable income has not fluctuated greatly. It shows a trend of increasing first, keeping stable in the medium term, and then declining. In the past two years, the economy has entered a new normal. Affected by the domestic economic slowdown and changes in the provincial economic structure, the income growth rate is slow [13]. The per capita consumption expenditure increased by 5.6 times from 3213.42 yuan in 2001 to 18145 yuan in 2020. In terms of the growth rate of consumption expenditure, the growth rate of consumption expenditure in 2004, 2007, 2011, and 2017 was rapid, while the growth rate in other years was small, and the growth rate was fluctuating. The per capita consumption expenditure and per capita disposable income showed a synchronous growth trend, but its growth rate was slightly lower than that of per capita disposable income.

#### 4.2.2. Average Marginal Propensity to Consume

**Figure 3:**
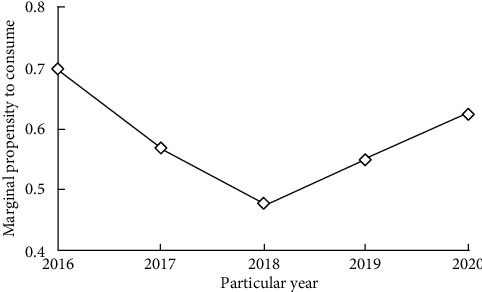
Marginal propensity to consume of Chinese household consumption structure.

Marginal propensity to consume refers to the proportion of increased consumption in increased income. According to the annual per capita consumption expenditure, per capita disposable income, increased consumption, and income of Chinese households from 2016 to 2020, the value of marginal propensity to consume is calculated, as shown in Figure 3.

According to the law of diminishing marginal propensity to consume, although people's consumption increases with the increase in income, the part used to increase consumption in the increased income is less and less. From 2016 to 2020, China's household marginal propensity to consume is within the limit of marginal propensity to consume (between 0 and 1), basically fluctuates between 0.5 and 1, and does not show an obvious decreasing trend, which does not conform to the law of diminishing marginal propensity to consume, indicating that China's household consumption confidence is insufficient [14]. In 2018, the marginal propensity to consume dropped to the lowest value of 0.47394, indicating that the consumer confidence of Chinese households was once in a high state. However, due to the slowdown of macro-economic growth, the aggravation of the subprime mortgage crisis, the increasing pressure of inflation, and other adverse factors, the marginal propensity to consume showed an upward trend at the beginning of 2008, which indicated that the consumer confidence of Chinese households was unstable in recent years.

### 4.3. Results of Structural Dynamic Analysis

#### 4.3.1. Structural Change of Consumption Expenditure

The change degree of consumption structure measures the change degree of residents' consumption structure every year. The larger the calculated value is, the greater the change range of residents' consumption structure in this period is. Combined with the consumption expenditure data of China in recent years, Chinese families are divided into three stages: 1997–1999, 2000–2013, and 2014–2020. These three stages correspond to the well-off, affluent, and richest life of Chinese families in turn. The specific statistics of structural change are shown in Table 3.

From the calculation results in Table 3, we can see that the average annual change of China's household consumption structure was 2.2 percentage points from 1997 to 1999, 0.73 percentage points from 2000 to 2013, and 3.73 percentage points from 2014 to 2020. According to the criteria for dividing social stages, the Engel coefficient was 45.9% in 1997 and 38.4% in 2000. At this stage, China has completed the transition from a well-off society to a relatively rich society [15]. Therefore, the change of consumption structure in this period is relatively large. From 2000 to 2013, China's household Engel coefficient was between 30% and 40%, showing a trend of first decline and then increase. Currently, the change of consumption structure is lower than that of the previous period. On the one hand, the change range of the previous period is large, which is difficult to maintain; on the other hand, it needs a long transition period from a well-off society to a more affluent society, and the residents' consumption structure is gradually improving, and the residents' rational consumption consciousness is also constantly improving. From 2014 to 2020, the Engel coefficient of Chinese families is less than 30%, and people's life has initially entered the richest stage. Therefore, the change of consumption structure in this period is relatively high.

#### 4.3.2. Elastic Analysis

According to the calculation principle of demand income elasticity, we can calculate the demand income elasticity of Chinese households' consumption expenditure. The demand income elasticity of food, clothing, housing, family equipment, medical treatment, transportation, culture and education, and other items are 0.763, 0.889, 0.958, 0.696, 0.721, 1.119, 0.648, and 0.842 respectively. Therefore, the demand income elasticity of residents' consumption is positive, which indicates that with the increase in disposable income, residents' consumption expenditure will increase accordingly.

Based on the analysis of basic consumption demand, marginal consumption tendency, and demand income elasticity of the ELES model, the basic consumption demand and marginal consumption tendency of food are in the first place; the marginal consumption propensity and demand income elasticity of transportation, communication, and housing are the largest, and the consumption of transportation, communication, and housing is developing into a hot area of urban residents' consumption in Hebei Province; people will pay more and more attention to medical treatment, culture, and education, and gradually change into the development type and enjoyment type of consumption [16].

## 5. Suggestions on Promoting the Optimization of China's Household Consumption Expenditure Structure

Consumption is of great significance in the development of national economy, and the current economic situation makes it particularly important to promote consumption. The government should optimize the structure of Chinese household consumption expenditure from many aspects.

### 5.1. Increasing the Income of Rural Residents through Various Ways

As far as the current economic situation is concerned, measures can be taken from the following aspects: first, accelerate the reform of state-owned enterprises, accelerate the improvement of the overall efficiency of state-owned enterprises, improve their economic efficiency and increase workers' wages; vigorously develop various forms of collective economy, encourage and support the healthy development of individual economy and private economy, create more employment opportunities, and increase residents' income. Support laid-off workers and other low-income people to ensure the minimum living standard of the city; encourage secondary entrepreneurship through technical training, policy support, financial assistance, etc., vigorously develop the individual and private economy, and gradually increase their income [17]. Encourage and support residents to broaden investment channels in various forms, such as investing in the stock industry; purchasing treasury bonds; and participating in insurance, real estate, education, tourism, and other industries, to realize the continuous appreciation of residents' assets. Finally, it is necessary to implement a moderate tax reduction policy for the individual economy and small- and medium-sized private economy. The administrative departments for industry and commerce and the health and epidemic prevention departments should increase their support, and establish corresponding support policies, to accelerate the prosperity of the individual and private economy and effectively improve the income of operators.

### 5.2. Guide Reasonable Consumption Concept

As the main body of consumption, consumers and consumption environment work together and promote each other. Therefore, it is necessary to guide consumers to consume reasonably and scientifically, change traditional consumption concepts, abandon the consumption habits of comparison and extravagance, and establish a correct and reasonable consumption concept. On the one hand, we need to improve the quality of consumers and update the concept of consumption through education and guidance, so that people can correctly understand the importance of scientific consumption mode to the harmonious development of human, society, and ecological environment. On the other hand, we should pay attention to the consumption of spiritual culture [18]. Complete material consumption is bound to fail to fully meet people's consumption needs. Therefore, the government needs to increase investment in spiritual culture, pay equal attention to spiritual consumption and material consumption, coordinate the development of the two, increase the proportion of Chinese education and entertainment in the consumption structure, improve the level of consumption, and further optimize the consumption structure, to effectively promote economic growth and achieve all-round social progress.

### 5.3. Optimize the Consumption Environment

As an external condition, consumption environment determines the utility that consumer goods can bring to consumers and then has an important impact on the consumption mode and behavior choice of residents. First, improve the consumer market laws and regulations. We should strengthen legal publicity, strengthen market supervision, ensure product quality, crack down on fake and shoddy commercial fraud, establish a good and orderly market order, and purify the consumption environment. To protect the legitimate consumption rights and interests of residents. Secondly, strengthen infrastructure construction [19]. Ensure the basic service conditions of power supply network, water supply network, information network, etc., according to the characteristics of different cities or regions, classify and make reasonable planning, and establish shopping places that meet the economic development level of Hebei Province [20, 21].

### 5.4. Expanding the Field of Consumption and Cultivating New Hot Spots of Consumption

In the process of adjusting the consumption structure of residents, in addition to the diversification and personalized supply of consumer goods in the traditional consumption field, we should continue to expand new consumption fields. According to the existing traditional market, we should find new potential consumption markets, such as information consumption and green consumption, to form new consumption hot spots and realize the sustainability of consumption structure optimization.

## 6. Conclusion

From the national level, according to the marginal propensity to consume, income elasticity of demand, and price elasticity of all kinds of consumer goods, we can know that the marginal propensity to consume food in China's household consumption expenditure structure is gradually decreasing, the marginal propensity to consume health care and housing is gradually increasing, and the household consumption expenditure structure is gradually changing to the development-type and enjoyment-type structure. From the regional level, due to different income and prices, there are differences between different provinces. Therefore, analyzing the results of residents' consumption expenditure can optimize the structure of residents' consumption expenditure and help rationalize the system of household consumption expenditure in all regions of China.

## Figures and Tables

**Table 1:** Types of household consumption expenditure structures in China.

Classification criteria	Types of consumption expenditure structures	Explain
The development level of consumption structure	Consumption structure of hunger and cold	Food consumption and clothing consumption are the focus, and the quality of food and clothing is poor; that is to say, the main part of consumption expenditure is used to meet the basic needs of life, and there are few development materials and enjoyment materials
	Food and clothing consumption structure	Food and clothing consumption structure is a medium-level consumption structure. Food consumption accounts for a large proportion of household consumption. When the income increases, the proportion of food consumption expenditure is basically unchanged, and the quality of food improves with the increase in income. The proportion of clothing consumption and housing consumption in household consumption is rising, and the quality of clothing and housing is improving. Consumption expenditure such as culture and services accounts for a small proportion
	Well-off consumption structure	The well-off consumption structure has reached a higher level. The proportion of food consumption in household consumption decreased significantly, the consumption expenditure of clothing and living decreased gradually, the consumption expenditure of medical and transportation increased continuously, and the consumption expenditure of culture and services increased significantly. The proportion of basic living consumption in household consumption is declining, mainly focusing on development materials and enjoyment materials
	Post well-off consumption structure	The proportion of food consumption expenditure in household consumption continued to decline, while the proportion of transportation and communication, education and culture, and service consumption expenditure increased significantly

**Table 2:** China's household consumption data from 2015 to 2020.

Project	Year					
	2015	2016	2017	2018	2019	2020
Disposable income	10129	11321	11759	13786	15781	19109
Consumption expenditure	7182	7943	8697	9997	11243	12265
Food	2710	2914	3112	3628	4260	4479
Clothing	687	801	902	1042	1166	1284
Live	733	809	904	983	1145	1229
Household appliances and services	407	446	498	602	692	787
Medical care	528	600	621	699	786	856
Traffic communication	844	997	1147	1358	1417	1683
Education, culture, and entertainment services	1033	1098	1203	1329	1358	1473
Other goods and services	240	278	309	358	418	474

**Table 3:** Changes of basic consumption structure of Chinese households.

	1997–1999 (%)	2000–2013 (%)	2014–2020 (%)
Household appliances and services	1.90	1.05	2.83
Medical care	1.39	0.83	0.93
Traffic communication	0.29	0.52	0.22
Education, culture, and entertainment	0.79	0.584	0.93
Live	0.68	0.59	0.41
Other goods and services	0.96	0.66	1.03
Changes in consumption structure	0.29	0.50	4.39
Average annual consumption structure change	0.30	0.41	0.45
Household appliances and services	6.60	5.10	11.18
Medical care	2.20	0.73	3.73

## Data Availability

The labeled dataset used to support the findings of this study is available from the corresponding author upon request.
